# Long-term outcome of CblC deficiency complicated with pulmonary hypertension

**DOI:** 10.1186/s13023-025-03839-5

**Published:** 2025-06-04

**Authors:** Si Ding, Yuxin Deng, Lili Hao, Wenjuan Qiu, Shengnan Wu, Yongxing Chen, Ting Chen, Xia Zhan, Lianshu Han, Xianting Jiao

**Affiliations:** 1 Department of Pediatric Endocrinology and Genetic Metabolism, Xinhua Hospital, Shanghai Institute of Pediatric Research, Shanghai Jiao Tong University School of Medicine, 1665 KongJiang Road, Shanghai, 200092 China; 2 Department of Endocrinology and Genetic Metabolism, Children’s Hospital Affiliated to Zhengzhou University, Henan Children’s Hospital, Zhengzhou, Henan, 400052 China; 3 Department of Pediatric Infection, Xinhua Hospital, Shanghai Jiao Tong University School of Medicine, 1665 KongJiang Road, Shanghai, 200092 China

**Keywords:** CblC deficiency, Methylmalonic acidemia, *MMACHC* gene, Pulmonary hypertension

## Abstract

**Objective:**

Pulmonary Hypertension (PH) in patients with cblC deficiency is one of the rare but lethal complications. This study aimed to described its characteristics and long-term outcome.

**Methods:**

A total of 26 patients with cblC deficiency complicated by PH were enrolled. Clinical and laboratory data were reviewed in detail.

**Results:**

Sixteen patients presented with PH manifestations as the initial symptom while ten patients developed PH after the involvement of other systems. The median onset age of PH was 3.25 years (ranging from 1 month to 13.4 years). Sixteen cases had other cardiovascular damage, including right cardiac enlargement, atrial septal defects, ventricular septal defects, left ventricular hypertrophy, pericardial effusion, pulmonary artery fistula and mild pulmonary artery stenosis. Intramuscular hydroxylcobalamin was given to all patients, together with L-carnitine, betaine and folinic acid after diagnosis. And PH targeted drags were given to 12 cases. As a result, propionylcarnitine, propionylcarnitine/acetylcarnitine ratio, methylmalonic acid, methylcitric acid and homocysteine levels decreased while methionine levels increased remarkably after treatment(*p*<0.05). The c.80 A> G variant was the most frequent allele in this cohort. Pulmonary artery systolic pressure was within the normal range in all patients, except that one case still had PH and two cases died. Multi-system involvement was improved overall.

**Conclusion:**

cblC deficiency should be considered in patients with PH. Multi-system evaluation, especially echocardiography is recommended to be performed regularly at each patient visit. The c.80 A > G variant might be a hot-spot mutation of cblC deficiency complicated with PH. Most patients show optimistic prognosis, while neurological damage is usually difficult to reverse.

## Introduction

Methylmalonic aciduria (MMA) is the most common inborn error of intracellular cobalamin metabolism. Based on the biochemical manifestation, MMA can be divided into two common types: isolated MMA and combined MMA and homocystinuria [1]. The cobalamin C (cblC) type of combined MMA and homocystinuria (cblC deficiency, OMIM 277400) caused by mutations in the *MMACHC* gene is the most common type of MMA in China [2]. This defect leads to the impaired synthesis of adenosylcobalamin and methylcobalamin, which are essential cofactors for the conversion of methylmalonyl-CoA into succinyl-CoA and homocysteine to methionine, respectively. As a result, homocysteine (HCY) and methylmalonic acid levels are elevated, accompanied by normal or decreased levels of methionine. Based on the age of onset, patients can be divided into two phenotypes, early-onset (onset <1 year) and late-onset (onset >1 year) [3]. The clinical manifestations of cblC deficiency are heterogeneous, mainly encompassing neurological, psychiatric, renal, ophthalmic and thromboembolic symptoms, of which neurological symptoms are the most common [4, 5]. Less widely known are cardiovascular diseases, which include structural heart diseases, pulmonary hypertension (PH), cardiomyopathy, hypertension and arrhythmias [6–8]. PH encompasses a group of disorders leading to elevated pressures within the pulmonary arteries. Currently, PH caused by metabolic disorders is classified in group 5, the prevalence of which was reported to be 5–20 cases per 10,000 in Australia [9]. PH in patients with cblC deficiency is one of the rare but lethal complications and unfortunately, diagnosis is often delayed or missed due to the heterogeneous manifestations. Also, limited research is available in its clinical characteristics and outcome.

In the present study, we performed a detailed retrospective chart review of clinical data in 26 patients with cblC deficiency complicated with PH. The aim of this research was to investigate the clinical and molecular characteristics of cblC deficiency complicated with PH and their long-term outcomes.

## Methods

### Patients

From 2006 to 2023, a total of 2352 patients with cblC deficiency were diagnosed at multiple hospitals in China. Among them, patients who developed PH and showed no improvement of PH without targeted treatment for cblC deficiency were included in this study. While those with PH secondary to infection, usually showing clinical improvement after two weeks of antibiotic therapy, were excluded. Their clinical characteristics, metabolites, molecular features and outcomes were reviewed. cblC deficiency was diagnosed by biochemical analysis and molecular analysis of *MMACHC* gene. The biochemical markers include increased levels of blood propionylcarnitine (C3), C3/acetylcarnitine (C2) ratio and HCY, urine methylmalonic acid and methylcitric acid and decreased or normal methionine levels [10]. The diagnosis of PH was based on pulmonary artery systolic pressure (PASP) which was above 40mmHg [11]. Patients presenting symptoms in the first year of life are defined as early-onset whereas those presenting symptoms at any time after one year are defined as late-onset [3]. Informed consent was obtained from the legal guardians of each patient. This study was approved by the Ethics Committee of Xinhua Hospital (approval No. XHEC-D-2024-033).

### Detection of metabolite

Amino acids and acylcarintines, including C3 and C2 were measured by tandem mass spectrometry (MS/MS; API 4000, American Systems Inc.) on dried blood filter papers. The ratios of C3/C2 were calculated at the same time [12]. Urinary organic acids including methylmalonic acid and methylcitric acid were detected by gas chromatography and mass spectrometry (GC/MS, QP2010, Shimadzu Corporation, Kyoto, Japan) [13]. Plasma HCY level was detected by fluorescence polarization immunoassay.

### *MMACHC* gene mutation analysis

Genomic DNA was extracted from peripheral blood samples. *MMACHC* gene test was performed by Sanger sequencing and high-throughput next-generation sequencing. The mutation was identified by the normal *MMACHC* sequence as a reference (NCBI GENEBANK, NM_015006.03). The ClinVar database, the HGMD database and the former literatures was used to identify whether the mutations had been reported.

### Echocardiography

A routine transthoracic echocardiography was performed on each child and PASP was assessed according to the measurement of tricuspid regurgitation velocity.

### Follow-up

Patients were followed up every 3–6 months, and the clinical evaluation mainly included their condition changes, recent treatment strategies as well as motor, speech, and cognitive development status. Amino acids, acylcarnitines and HCY levels in blood and organic acids levels in urine were analyzed regularly. Blood routine examination, liver function, echocardiography examinations, and brain magnetic resonance imaging (MRI) were also monitored.

### Statistical analyses

Statistical analyses were performed by SPSS 26.0 (IBM Corp., Armonk, New York). Continuous variables are presented as the mean ± SD or median (range). Data that did not significantly deviate from normal distribution were tested using an unpaired two-tailed t-test, and non-normally distributed data were tested using the Mann-Whitney U test. Categorical variables are presented as percentages (%).

## Results

### Clinical manifestation

The detailed information on patients diagnosed as cblC deficiency complicated with PH is summarized in Table 1. A total of 26 patients were recruited in our cohort, including 15 males and 11 females. Early-onset form was observed in 15 cases (58%), while late-onset form was observed in 11 cases (42%). The median age of cblC deficiency and PH symptoms onset was 5 months (range 1 day − 13.5 years old) and 3.25 years (range 1 month − 13.5 years old), respectively. Sixteen patients had PH manifestations as the initial symptom, among whom seven were early-onset form and nine were late-onset form. Ten patients developed PH symptoms after the involvement of other systems. Among them, eight patients with early-onset form, presented symptoms such as jaundice, anemia, feeding difficulties, and developmental delay within the half year of life. The onset interval between cblC deficiency and PH ranged from 2 months to 8.5 years. Two late-onset cases had acute renal failure and cognitive decline as first presenting symptom, respectively, with PH symptoms occurred 2 and 9 months later (P16, P23). The main presentations of PH included tachypnea/dyspnea (76.9%), fatigue/ decreased activity tolerance (23.1%), cyanosis of lips (19.2%), edema of lower limbs (11.5%) and fainting (11.5%). And a total of 11 cases (42.3%) had fever or cough prior to disease onset.

**Table 1:** Clinical characteristics of the patients with CblC deficiency complicated by PH

No	Sex	Onset age of cblC deficiency (year)	Onset age of PH (year)	Initial symptoms	Symptoms due to PH	Other cardiovascular involvement	Other systems involvement	PASP (mmHg)	Treatment	
									OHCbl dosage (mg/kg/day)	PH targeted drugs
1	M	0.4	0.6	Lethargy, feeding difficulties	Tachypnea	Atrial septal defects	Anemia, mental retardation	88	0.11	Bosentan
2	M	0.3	0.6	Jaundice, anemia	Tachypnea		Convulsion	-	0.16	
3	M	0.3	0.3	PH manifestations	Dyspnea	Right cardiac enlargement	Developmental delay	-	0.14	
4	M	3.0	3.0	PH manifestations	Tachypnea		Proteinuria	-	0.02	Bosentan
5	F	4.3	4.3	PH manifestations	Fainting	Right cardiac enlargement		41	0.11	Bosentan, sildenafil
6	F	0.3	0.3	PH manifestations	Tachypnea, elevated heart rate	Atrial septal defects, pulmonary artery fistula		44	0.20	
7	M	After birth	0.1	PH manifestations	Dyspnea	Ventricular septal defects	Jaundice, granulopenia, coagulopathy, acute renal failure	-	0.49	
8	F	0.3	4.2	Developmental delay	Cyanosis of lips, tachypnea, edema of lower limbs			62	0.21	Bosentan,
9	M	0.1	4.2	Jaundice	Tachypnea, cyanosis of lips	Pericardial effusion	Anemia	80	0.48	Bosentan, sildenafil
10	F	13.4	13.4	PH manifestations	Tachypnea, decreased activity tolerance	Right cardiac enlargement		-	0.03	Bosentan, tadalafil
11	M	5.0	5	PH manifestations	Fatigue, tachypnea	Right cardiac enlargement		50	0.09	Bosentan, sildenafil
12	M	11.0	11	PH manifestations	Tachypnea, decreased activity tolerance, elevated heart rate, fainting	Right cardiac enlargement		57	0.07	
13	F	After birth	0.2	Feeding difficulties, failure to thrive	Dyspnea		Developmental delay, seizure, anemia	64	0.56	sildenafil
14	M	7.6	7.6	PH manifestations	Tachypnea	Right cardiac enlargement	Proteinuria	55	0.14	
15	M	0.2	0.2	PH manifestations	Tachypnea, edema of lower limbs	Atrial septal defects	Hematuria	58	0.57	Bosentan, sildenafil
16	M	10.1	10.1	Feeding difficulties, headache, vomiting	Fatigue	Left ventricular hypertrophy	Chronic renal failure, thrombotic microangiopathy	-	0.08	
17	F	0.2	1.2	Feeding difficulties, jaundice	Cyanosis of lips, tachypnea	Right cardiac enlargement	Anemia	-	0.10	Bosentan
18	M	5.0	5	PH manifestations	Fatigue	Right cardiac enlargement		94	0.22	
19	F	0.1	0.1	PH manifestations	Tachypnea	Ventricular septal defects, Left ventricular hypertrophy mild pulmonary artery stenosis		54	0.55	Bosentan
20	F	0.8	0.8	PH manifestations	Tachypnea			-	0.10	Sildenafil
21	M	3.5	3.5	PH manifestations	Edema of lower limbs			-	0.08	
22	M	4.0	4	PH manifestations	Cyanosis of lips, fainting			-	0.06	
23	F	11.2	12	Cognitive decline	Fatigue, tachypnea		Motor impairment	76	0.07	Bosentan, sildenafil
24	F	0.2	0.2	PH manifestations	Tachypnea			-	0.47	
25	M	0.5	0.8	Anemia	Cyanosis of lips		Hydrocephalus	60	0.27	
26	F	0.3	10	Developmental delay	Tachypnea, fatigue	Atrial septal defects		60	0.29	

PH, Pulmonary hypertension; PASP, pulmonary artery systolic pressure; OHCbl, hydroxylcobalamin

-,no data

Overall, 24 patients had other systems being involved. Among them, neurological symptoms, encompassing convulsion, developmental delay, mental retardation and movement disorder, were present in 12 patients. Hematological system disorders, including anemia, granulocytopenia and thrombocytopenia, were observed in 11 patients. Digestive system involvement consisting of jaundice, vomiting and feeding difficulties occurred in ten patients. Kidney involvement manifesting as hematuria, proteinuria and renal thrombotic microangiopathy was presented in five patients.

### Biochemical characteristics

Biochemical markers of patients with cblC deficiency were measured before and after treatment. As is shown in Table 2, the levels of methylmalonic acid and HCY in all patients were above the cutoff values accompanied by normal or elevated levels of C3, C3/C2 ratios and methylcitric acid and normal or decreased levels of methionine before treatment. And at the final follow-up, concentrations of blood C3, C3/C2 ratio and HCY as well as urine methylmalonic acid and methylcitric acid showed remarkably decrease tendency while blood methionine levels increased greatly, with a significant statistical difference (*P* < 0.05).

**Table 2:** Comparison of biochemical data of patients in blood and urine before and after treatment

	C3(µmol/L)		C3/C2		Methionine (µmol/L)		Methylmalonic acid (mmol/mol cr)		Methylcitric acid (mmol/mol cr)		HCY (µmol/L)	
	*n*	Median (range)	*n*	Median (range)	*n*	Median (range)	*n*	Median (range)	*n*	Median (range)	*n*	Median (range)
BEFORE TREATMENT	21	7.96 (2.02–25.41)	20	0.32 (0.07–1.20)	17	7.86 (2.94–30.40)	18	64.55 (8.62-288.61)	16	2.10 (0.00-16.53)	25	136.04 (50.00-779.40)
AFTER TREATMENT	20	4.20 (2.09–9.23)	19	0.16 (0.02–0.33)	16	21.51 (10.76–58.64)	17	1.80 (0.00-83.30)	15	0.10 (0.00-4.01)	22	30.55 (11.60-69.26)
P VALUE	**0.004**		**0.005**		**0.011**		**0.007**		**0.028**		**<0.001**	
REFERENCE RANGE	0.40-4.00		0.03–0.20		9.00–45.00		0.00–4.00		0.00-0.70		<15.00	

### Echocardiographic features

Echocardiography was performed on all patients, and the estimated PASP of 15 patients with specific values ranged from 41-94mmHg, with a median value of 60 mmHg and an average value of 62 mmHg (some patients who were diagnosed in other hospitals only had the result of echocardiography without specific value). Additionally, other types of cardiovascular involvement were occurred in 16 patients, including right cardiac enlargement (8 cases), atrial septal defects (4 cases), ventricular septal defects (2 cases), left ventricular hypertrophy (2 cases), pericardial effusion (1 case), pulmonary artery fistula (1 case) and mild pulmonary artery stenosis (1 case).

### Molecular analysis

All 26 patients underwent genetic testing and details are shown in Table 3. One patient was homozygote and the remaining 25 patients were compound heterozygotes. A total of 12 known *MMACHC* mutations were identified, including four frameshift mutations, three missense mutations, three nonsense mutations and two deletion variations. The most frequent variant in this cohort is c.80 A > G, found in 18 cases (69.2%).

**Table 3:** *MMACHC* variants in 26 patients with CblC deficiency complicated by PH

No	Allele 1			Allele 2		
	Nucleotide change	Protein change	Exon	Nucleotide change	Protein change	Exon
1	c.80 A > G	p.Q27R	1	c.609G > A	p.W203X	4
2	c.80 A > G	p.Q27R	1	c.609G > A	p.W203X	4
3	c.80 A > G	p.Q27R	1	c.609G > A	p.W203X	4
4	c.80 A > G	p.Q27R	1	c.658_660delAAG	p. K220del	4
5	c.80 A > G	p.Q27R	1	c.637G > T	p.E213X	4
6	c.80 A > G	p.Q27R	1	c.440_441delGT	p.C149Hfs*32	4
7	c.80 A > G	p.Q27R	1	c.567dupT	p. I190Yfs*13	4
8	c.80 A > G	p.Q27R	1	c.609G > A	p.W203X	4
9	c.80 A > G	p.Q27R	1	c.609G > A	p.W203X	4
10	c.482G > A	p.R161Q	4	c.658_660delAAG	p. K220del	4
11	c.80 A > G	p.Q27R	1	c.609G > A	p.W203X	4
12	c.452 A > G	p.H151R	4	c.609G > A	p.W203X	4
13	c.609G > A	p.W203X	4	c.658_660delAAG	p. K220del	4
14	c.80 A > G	p.Q27R	1	c.609G > A	p.W203X	4
15	c.80 A > G	p.Q27R	1	c.615 C > A	p.Y205X	4
16	c.482G > A	p.R161Q	4	c.658_660delAAG	p. K220del	4
17	c.80 A > G	p.Q27R	1	c.452 A > G	p.H151R	4
18	c.80 A > G	p.Q27R	1	c.658_660delAAG	p. K220del	4
19	c.271dupA	pR91fs	2	c.609G > A	p.W203X	4
20	c.445_446delTG	p.C149Hfs*32	4	c.609G > A	p.W203X	4
21	c.80 A > G	p.Q27R	1	c.658_660delAAG	p. K220del	4
22	c.80 A > G	p.Q27R	1	c.609G > A	p.W203X	4
23	c.482G > A	p.R161Q	4	c.609G > A	p.W203X	4
24	c.658_660delAAG	p. K220del	4	c.658_660delAAG	p. K220del	4
25	c.80 A > G	p.Q27R	1	c.567dupT	p. I190Yfs*13	4
26	c.80 A > G	p.Q27R	1	c.609G > A	p.W203X	4

### Treatment

All patients maintained a normal diet without protein restriction. Generally, long-term treatment contained hydroxylcobalamin at a dose of 5 to 20 mg, intramuscular injected daily to weekly (detailed dosage is shown in Table 1), supplemented with oral drugs including L-carnitine at 50–100 mg/kg/day, betaine at 100–300 mg/kg/day and folinic acid at 5–15 mg/day. After the onset of PH, patients were treated with oxygen therapy, diuretic and cardiotonics according to their condition. A total of 12 patients were given targeted PH drugs. Among them, five received bosentan, two received sildenafil, five received bosentan and sildenafil, and one received bosentan and tadalafil. The rest did not accept treatment with targeted PH drugs.

### Follow-up and clinical outcome

At the final follow-up, two cases died (P7and P11) due to multiple organ failure and severe heart failure, respectively, two cases were lost to follow-up (P2 and P4) and the remaining 22 cases were alive with a 0.5–7.3 years follow-up. The PASP of all children was within the normal range and no obvious abnormality was found in cardiac ultrasound, except one case (P22) still had PH at half of a year follow-up and received treatment with oral Bosentan. As for other sequelae, the involvement of hematological system and digestive system were recovered while neurological symptoms and kidney involvement were improved, yet seven cases suffered varying degrees of intellectual or motor impairment and one patient remained chronic renal failure.

## Discussion

cblC deficiency is the most common disorder of organic acidemia in China, while PH as a leading symptom is rare and these cases have mainly been presented with case reports. Thus, this study aimed to elucidate the clinical data of 26 patients with cblC deficiency complicated by PH and evaluate their long-term outcome, which is valuable for physicians who encounter such cases.

In this study, 57.7% (15/26) of the patients presented with early-onset. This inconsistent with that described in the previous cases where cblC deficiency with PH is more common in late-onset patients [14–16]. This difference might be due to the fact that PH manifestations were mainly presented as the initial symptom in the previous case reports. In our study, except 16 patients who had PH manifestations as the initial symptom (seven cases with early-onset and nine cases with late-onset), there were 10 patients who developed PH after other symptoms occurred (eight cases with early-onset and two cases with late-onset). This suggests that PH manifestations often appear as an initial symptom in patients with cblC deficiency, particularly for late-onset cases, however, it can also present in early-onset patients after the involvement of other systems.

In the present study, patients with cblC deficiency mainly manifested as tachypnea/dyspnea, fatigue/ decreased activity tolerance, cyanosis of lips, edema of lower limbs and fainting when developing PH. A total of eleven patients had fever or cough prior to disease onset and two patients died during treatment. All these findings are consistent with previous study [15, 17]. This suggests that patients with cblC deficiency complicated with PH commonly present with symptoms of hypoxia and infection is considered to be the trigger. And in serious conditions, this defect tends to be life-threatening.

In previous cases, patients with cblC deficiency complicated with PH often presented with involvement of other systems, with kidney involvement being the most common [15, 17]. Mild cases may present with hematuria or proteinuria, while severe cases can develop hemolytic uremic syndrome or even renal failure, with renal pathology of reveal thrombotic microangiopathy. The pathophysiologic mechanisms may involve impaired mitochondrial energy metabolism and endothelial cell damage induced by methylmalonic acid and HCY [3, 18]. In our cohort, 24 patients had other systems being involved. Among them, neurological symptoms were frequently observed, whereas kidney involvement occurred less commonly. The difference might be related to genetic predisposition, environmental influences and dietary patterns [19], though single-center data limitations should also be considered.

Metabolite analysis using MS/MS combined with GC/MS is important for the diagnosis of MMA and determination of vitamin B12 effectiveness. Plasma HCY in patients with cblC deficiency is elevated remarkably so the HCY assay provides a rapid and simple method for clinical diagnosis [16, 20]. The mechanism whereby cblC deficiency can cause PH is unelucidated. It has been reported that HCY > 45µmol/L is related to the development of vascular complications [21]. High HCY levels may inhibit the synthesis and release of nitric oxide and increase oxidative stress, impairing vasodilation, stimulating and proliferating of vascular smooth muscle cells, thus leading to vascular damage and remodeling. In addition, it can also cause thrombosis by disrupting coagulation and fibrinolysis balance in the body [3, 22–24]. Although high levels of HCY seem to be related to PH, in some diseases with elevated HCY levels (not accompanied by elevated methylmalonic acid levels) such as cystethionine synthase deficiency, PH does not usually occur. In addition, there are some case reports of isolated MMA complicated with PH [14, 25, 26], which might be due to the dysfunction of mitochondrial functional synthesis and endothelial cell damage caused by elevated methylmalonic acid [27]. Elevated levels of HCY and methylmalonic acid are typic hallmarkers of cblC deficiency. However, in our center, 2,352 patients were diagnosed as cblC deficiency while only 26 patients presented with PH, the others did not present with PH during the long-term follow up. This suggests that the pathogenic factors of PH might more than the increasing levels of HCY and methylmalonic acid, other accumulated toxic metabolites might also work. In our cohort, patients appeared to be beneficial to the treatment yet the biochemical markers still beyond the normal range in most patients, especially for HCY. Further study needs to be conducted to determine whether it has a long-term effect on blood vessels.

Genetic analysis is an important tool for clarifying diagnosis, prenatal diagnosis and genetic counseling. Until now, more than 100 *MMACHC* gene mutations have been reported and there might be a correlation between phenotype and genotype [28–30]. Although c.609G> A was reported to be the most common variant in Chinese population [2], in the present study, the c.80 A > G variant was observed the most frequently in our cohort, which is consistent with previous study [15]. This suggests that c.80 A > G might be a hot spot mutation of cblC deficiency complicated with PH in Chinese population. However, a total of 297 patients carried c.80 A > G among the 2352 patients with cblC deficiency, and none of them developed PH except the 18 cases. Further study needs to determine whether another gene shows synergism with *MMACHC* gene, leading to the occurrence of PH. While in Europe, c. 271dupA, c.276G > T, c.464G > A and c.484G > T were reported to be associated with cblC deficiency complicated with PH [17, 31–33]. One of the probable reasons might be associated with ark effect and genetic drift [34]. In addition, cases of PH caused by other combined MMA have not been reported so far. It might be related to the low incidence of other combined MMA and whether *MMACHC* gene mutations have specific vascular pathogenicity needs to be clarified further.

The therapeutic goal of cblC deficiency is to improve clinical symptoms and biochemical markers. For patients with cblC deficiency, diet restriction is not necessary. Intramuscular injections of hydroxocobalamin have been proved to be beneficial and oral drugs supplementation including betaine, L-carnitine and folinic acid are used as an adjunctive therapy [4, 10]. For patients with PH, appropriate treatment such as oxygen inhalation, cardiotonic and diuresis should be given according to the condition, and targeted PH therapy is helpful to improve the survival rate of patients with idiopathic and hereditary PH [8, 35]. In our study, most patients appeared to be beneficial to the cblC deficiency treatment with or without PH-targeted therapy, showing improvement of clinical symptoms and biochemical indicators. It should be notable that without the essential treatment of cblC deficiency, PH-targeted therapy alone has limited effect [36], which indicates the important of correct diagnosis of cblC deficiency as soon as possible.

The experience of the diagnosis and treatment of cblC deficiency in our institution tends to be reprehensive in China, since our team possesses the largest cohort of patients with cblC deficiency and have done many relative researches. However, there are some limitations in this study. The first is the retrospective essence of this study. Patients were assessed in different hospitals during the follow-up, which led to the varying degrees of follow-up. Next, some patients were adopted with telephone follow-up to do an assessment, preventing comprehensive data collection. Last but not least is the relatively small sample size and the data in this study only applies to patients who had cblC deficiency combined with PH, which cannot be extended to other patients with related lesions. There may be differences in clinical characteristics and prognosis in these patients.

In conclusion, PH manifestations often appear as the first symptom of cblC deficiency, which is common in late-onset patients, while patients with early-onset may also develop PH after the involvement of other systems. Anoxic symptoms such as tachypnea/dyspnea, fatigue/ decreased activity tolerance and cyanosis of lips are common manifestations of PH in patients with cblC deficiency, which are often induced by infection. *MMACHC* gene c.80 A > G may be a hot spot mutation of this disease. Prompt treatment of cblC deficiency and targeted treatment of PH can effectively improve clinical symptoms and biochemical indicators, but the neurological damage is usually difficult to reverse. Critically ill patients can also be life-threatening. Thus, cblC deficiency should be considered in patients with multiple organ damage. Also, multi-system evaluation should be performed regularly in the diagnosis and treatment of cblC deficiency.

## Data Availability

All data generated or analyzed during this study are included in this published article and its supplementary information files.

## Abbreviations

PH: Pulmonary hypertension
PASP: Pulmonary artery systolic pressure
MMA: Methylmalonic acidemia
cblC: Cobalamin C
HCY: Homocysteine
C3: Propionylcarnitine
C2: Acetylcarnitine
C3/C2: Propionylcarnitine to acetylcarnitine ratio
MS/MS: Tandem mass spectrometry
GC/MS: Chromatography/mass spectrometry
